# Clinical characteristics and molecular genetic analysis of 73 cases of tuberous sclerosis complex caused by *TSC1/2* gene mutations in children

**DOI:** 10.3389/fped.2026.1795318

**Published:** 2026-04-13

**Authors:** Xiao Wu, Ming-Ying He, Hai-Feng Xu, Yan He, Xiao-Hui Min, Qi-Kai Zhao, Shu-Qi Liang, Nooraynee Bibi Needah Ginowree, Shu-Han Hu, Jia Yin, Zi-Han Fan, M. Jalal Ud Din, Min Zhu, Gang Zhang

**Affiliations:** 1 Department of Neurology, Children’s Hospital of Nanjing Medical University, Nanjing, Jiangsu, China; 2 Department of Pediatrics, Peking University Shenzhen Hospital, Shenzhen, Guangdong, China; 3 Nanjing Medical University, Nanjing, Jiangsu, China; 4 Department of Rehabilitation Medicine, Children’s Hospital of Nanjing Medical University, Nanjing, Jiangsu, China

**Keywords:** clinical features, epilepsy, molecular genetics, *TSC1/TSC2*, tuberous sclerosis complex

## Abstract

**Objective:**

This study analyzed the clinical and genetic characteristics of 73 pediatric patients with tuberous sclerosis complex (TSC). Through an examination of genotype-phenotype correlations, the research aimed to identify patterns in mutation characteristics to facilitate the optimization of diagnostic, therapeutic, and prognostic strategies.

**Methods:**

This retrospective study analyzed pediatric patients with TSC at Nanjing Medical University Children's Hospital between February 2018 and June 2025. Clinical data, including demographics and initial manifestations, were reviewed and peripheral blood samples were collected for whole-exome sequencing. Statistical analysis of categorical variables was performed using the chi-square test.

**Results:**

Among the 73 pediatric TSC patients, 62 (85%) were diagnosed with epilepsy, with seizures being the initial manifestation in 56 (90%) of these cases. The observed seizure types included epileptic spasms (*n* = 25), generalized tonic-clonic seizure (*n* = 13), focal impaired consciousness seizure (*n* = 10), focal preserved consciousness seizure (*n* = 6), focal to bilateral tonic-clonic seizure (*n* = 5), tonic (*n* = 1), and absence (*n* = 1). Other common clinical features were hypopigmented macules (*n* = 44), cortical tubers (*n* = 34), intellectual disability (*n* = 24), and subependymal nodules (*n* = 22). Genetic testing identified *TSC1* or *TSC2* mutations in 68 patients (93%), corresponding to 71 distinct mutation sites. Fourteen variants (2 in *TSC1*, 12 in *TSC2*) were novel. The spectrum of mutations included nonsense, frameshift, missense, and splice-site types, with both *de novo* and inherited origins identified.

**Conclusion:**

The clinical phenotype of TSC is highly heterogeneous, with complex genotype-phenotype associations. The identification of 14 novel variants expands the known mutational spectrum of TSC, and the detailed genotype–phenotype analysis provides valuable insights for early diagnosis, genetic counseling, and personalized therapeutic strategies in pediatric populations. Early *TSC1/TSC2* genetic testing is therefore crucial for diagnostic confirmation and enables personalized management strategies in cases of suspected TSC.

## 1 Introduction

Tuberous Sclerosis Complex (TSC), also known as Bourneville disease, is an autosomal dominant neurocutaneous syndrome affecting multiple organs including the skin, brain, heart, kidneys, and lungs (1). TSC has an estimated incidence of 1 in 6,000–1 in 10,000 live births (2), with no significant racial predilection. Approximately one-third of the cases are familial, whereas two-thirds are sporadic. While the proportions of *TSC1* and *TSC2* mutations are comparable in familial cases, *TSC2* mutations predominate in sporadic patients. The disorder stems from pathogenic mutations that disrupt the normal development of ectodermal, mesodermal, and endodermal lineages. Virtually any organ system can be involved, resulting in a highly complex and variable phenotype, with neurological and cutaneous manifestations being the most common (3). Approximately 30%–40% of patients with TSC exhibit Vogt's triad, characterized by epileptic seizures, facial angiofibromas, and intellectual disability (4–6). Other common clinical features include hypopigmented macules, cardiac rhabdomyomas, renal cysts, and retinal hamartomas. The clinical presentation varies with age in pediatric patients. Cardiac rhabdomyomas are most prevalent in fetal and neonatal population. From infancy through adolescence, commonly observed features include epilepsy, hypopigmented macules, and multiple intracranial calcifications. TSC is caused by loss-of-function mutations in the tumor suppressor genes *TSC1* or *TSC2*. The *TSC1* gene is responsible for encoding the protein hamartin, while the *TSC2* gene gives rise to the protein tuberin (7, 8). These proteins form a heterodimeric complex in the cytoplasm that functions as a GTPase-activating protein (GAP) toward Rheb, thereby negatively regulating the mammalian target of rapamycin (mTOR) signaling pathway (9). Mutations disrupting hamartin or tuberin synthesis lead to constitutive activation of the mTOR pathway. This dysregulation promotes aberrant cellular growth and proliferation, resulting in the formation of hamartomas in multiple organs and associated functional impairments. Given the critical role of mTOR signaling in neuronal development, its hyperactivation underlies the neurological manifestations of TSC, such as epilepsy and intellectual disability (10, 11). Pathogenic mutations in *TSC1* and *TSC2* are heterogeneous, with no predominant hotspot identified. Currently, *TSC1* mutations are predominantly nonsense or frameshift variants, whereas *TSC2* mutations more frequently involve missense changes or large deletions.

Although several cohort studies on tuberous sclerosis complex have been reported in China, most have focused on mixed-age populations or were confined to specific clinical phenotypes. To date, there have been few comprehensive clinical and genetic characterizations of exclusively pediatric TSC cohorts using whole-exome sequencing. This diagnostic complexity is compounded by age-dependent manifestations, such as cardiac rhabdomyomas, which typically appear in infancy and often regress spontaneously, whereas renal angiomyolipomas tend to emerge later in childhood or adolescence. Meanwhile, the marked phenotypic heterogeneity of TSC poses a challenge to clinical diagnosis. This challenge is especially urgent in pediatric populations, where epilepsy—often refractory—affects up to 90% of patients. Recurrent seizures in these individuals frequently result in irreversible neurocognitive deficits, such as intellectual disability (12). Consequently, the elucidation and demonstration of robust genotype-phenotype correlations is of critical importance. In this study, we present one of the largest single-center pediatric TSC cohorts in China, comprising 73 patients systematically evaluated by whole-exome sequencing. Our results expand the known mutational and phenotypic spectrum of disorders related to *TSC1* and *TSC2* offering insights that can aid in prenatal diagnosis, guide clinical screening, and optimize therapeutic approaches. Ultimately, this work advances the overall comprehension of TSC pathogenesis and clinical management.

## 2 Methods

### 2.1 Study subjects

We conducted a retrospective review of pediatric TSC patients at Nanjing Medical University Children's Hospital between February 2018 and June 2025, encompassing both inpatient and outpatient visits. Eligible subjects were identified using predefined inclusion and exclusion criteria. The study protocol was approved by the hospital's Institutional Review Board.

Since patient enrollment for this study began in February 2018, prior to the publication of the updated 2021 guidelines, the diagnostic criteria from the 2012 International Tuberous Sclerosis Complex Consensus Conference were applied. This approach was taken to ensure diagnostic consistency throughout the entirety of the retrospective analysis. It is important to note that a subsequent review confirmed that every patient included in the study also satisfies the 2021 criteria, as the fundamental clinical features used for diagnosis have remained largely unchanged between the two versions (13).

Inclusion Criteria: (1) Fulfill the 2012 International Tuberous Sclerosis Complex Consensus Conference diagnostic criteria, confirmed by the presence of either two major features or one major feature plus two minor features. (2) Provide voluntary assent (with parental/guardian consent) and written informed consent, with demonstrated good compliance and ability to cooperate with follow-up procedures. (3) Age < 18 years at enrollment.

Exclusion Criteria: Poor family cooperation, lack of collaboration, or missing clinical data;.

### 2.2 Materials and methods

#### 2.2.1 Clinical data collection

We retrospectively analyzed 73 consecutive pediatric patients with TSC treated at Nanjing Medical University Children's Hospital from February 2018 to June 2025. Data collection encompassed detailed medical history, physical examination findings, and results of diagnostic investigations. Required imaging for all patients included head CT and/or MRI. Additional assessments included cardiac echocardiography, renal and abdominal ultrasonography, fundus examination, and, in select cases, whole-exome sequencing. We systematically documented the following variables: demographic characteristics (sex, age at disease onset, and age at presentation), clinical features (reason for initial presentation, chief complaint, and phenotype), past medical and family history, results from all ancillary tests and imaging studies, final diagnosis, and treatment regimen.

#### 2.2.2 Genetic testing and analysis

Genetic testing was performed on peripheral blood samples from all 73 patients using whole-exome sequencing (WES). All identified *TSC1/TSC2* variants were validated by Sanger sequencing through pedigree analysis. The sequences were aligned to the human genome reference sequence (GRCh37/hg19) from the UCSC database and compared with the known *TSC1/TSC2* sequences (NCBI transcript numbers: NM_000368 for *TSC1*, NM_000548 for *TSC2*). Variants were classified as pathogenic, likely pathogenic, or variants of uncertain significance according to the American College of Medical Genetics and Genomics (ACMG) guidelines.

#### 2.2.3 Statistical methods

Statistical analysis was performed using SPSS software (version 25.0). Categorical data are reported as number (percentage). The chi-square test was employed to assess differences in phenotypic and genotypic traits across patient groups, with a *P*-value < 0.05 deemed statistically significant.

## 3 Results

### 3.1 Genetic characteristics

Genetic testing was performed on all 73 pediatric patients with TSC. Pathogenic mutations in either the *TSC1* or *TSC2* gene were identified in 68 patients (93%); no mutation was detected in the remaining five cases. A total of 71 distinct TSC1/2 mutation sites were found. These comprised 19 mutations in TSC1 and 52 in TSC2. One patient harbored two concurrent TSC2 mutations, and another had three concurrent TSC2 mutations. All other patients carried a single heterozygous mutation. All mutations were heterozygous.

**Figure 1:**
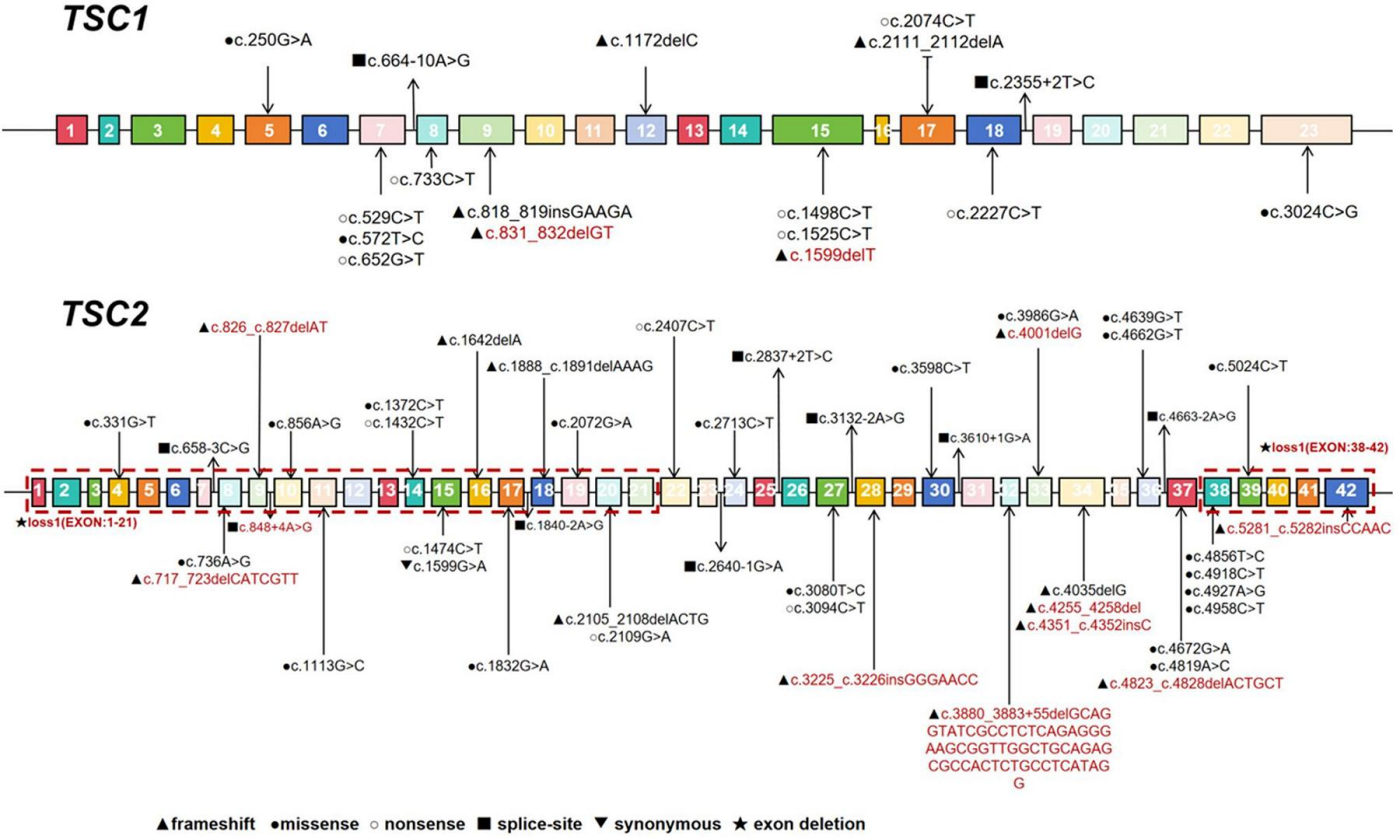
This study analyzed the *TSC1* and *TSC2* genes in 73 pediatric patients with tuberous sclerosis complex (TSC). Among the 68 mutation-positive cases, a total of 71 distinct mutation sites were identified. The mutational spectrum differed between genes: nonsense (9/19, 47%) and frameshift (5/19, 26%) mutations predominated in *TSC1*, whereas the *TSC2* spectrum was more diverse, with missense (22/52, 42%) and frameshift (13/52, 25%) mutations being most common. The figure illustrates all detected variant sites within the schematic structures of the *TSC1* (upper panel) and *TSC2* (lower panel) genes. Numbered boxes represent individual exons. Mutation types are distinguished by symbols: ● missense, ○ nonsense, ▴ frameshift, ▪ splice-site, ▾ synonymous, and ★ exon deletion. Novel variants identified in this study are highlighted in red.

The *TSC1* gene exhibited 11 *de novo* mutations and 8 inherited mutations, including 4 paternal and 4 maternal variants. Among the 19 *TSC1* gene mutations, 9 were nonsense mutations, 5 were frameshift mutations, 3 were missense mutations, and 2 were splice site mutations. Among the 52 *TSC2* gene mutations, 29 were *de novo* variants, 21 were inherited variants (8 paternal, 13 maternal), and 2 had unknown origins. The 52 *TSC2* gene mutations comprised 22 missense mutations, 13 frameshift mutations, 6 nonsense mutations, 8 splice site mutations, 1 synonymous mutation, and 2 exon deletions (Figure 1).

In this study, we identified 14 previously unreported mutation sites. Two were in the *TSC1* gene: c.1599delT and c.831_832delGT. The remaining 12 were in *TSC2*: c.3225_3226insGGGAACC, c.826_827delAT, c.4255_4258del, c.3880_3883 + 55del, c.5281_5282insCCAAC, c.4351_4352insC, c.4823_4828delACTGCT, c.717_723delCATCGTT, c.848 + 4A > G, c.4001delG, a heterozygous deletion of exons 38–42, and a heterozygous deletion of exons 1–21 (Table 1).

**Table 1:** Distribution of novel mutations identified in the TSC1/TSC2 gene.

Gene	Location	Nucleotide change	Protein change	Classification	Evidence of pathogenicity	Family History
Point Mutation of *TSC1*	Exon9	c.831 _832 delGT	p.His279Profs*20	Frameshift	P	*De novo*
	Exon15	c.1599delT	p.Glu534fsTer22	Frameshift	P	*De novo*
Point Mutation of *TSC2*	Exon8	c.717_723delCATCGTT	p.Phe239Leufs*19	Frameshift	LP	Maternal
	Exon9	c.826_c.827delAT	p.Met276Valfs*61	Frameshift	P	*De novo*
	Exon28	c.3225_c.3226insGGGAACC	p.Thr1077fsTer93	Frameshift	P	*De novo*
	Exon30	c.3880_c.3883 + 55delGCAGGTATCGCCTCTCAGAGGGAAGCGGTTGGCTGCAGAGCGCCACTCTGCCTCATAGG	p.Ala1294PhefsTer30	Frameshift	LP	Maternal
	Exon33	c.4001delG	p.Ser1334Thrfs*49	Frameshift	P	*De novo*
	Exon34	c.4255_c.4258del	p.Ser1420GlyfsTer55	Frameshift	LP	Maternal
	Exon34	c.4351_c.4352insC	p.Arg1451fsTer73	Frameshift	P	*De novo*
	Exon37	c.4823_c.4828delACTGCT	p.Tyr1608_Cys1609del	Frameshift	LP	*De novo*
	IVS9	c.848 + 4A > G	/	Splicing	VUS	Maternal
	Exon42	c.5281_c.5282insCCAAC	p.Ser1761Serfs*67	Frameshift	LP	Maternal
	Exon1–21	/	/	Deletion	P	*De novo*
	Exon38–42	/	/	Deletion	P	Unknown

All variants were classified according to the American College of Medical Genetics and Genomics (ACMG) guidelines.

RefSeq: TSC1: NM_000368; TSC2: NM_000548.

TSC, tuberous sclerosis complex; P, pathogenic; LP, likely pathogenic; VUS, variants of uncertain significance; RefSeq, reference sequence.

### 3.2 Clinical characteristics

This study included 73 pediatric patients with TSC, all of Han Chinese ethnicity. The cohort included 46 males and 27 females (male-to-female ratio, 1.70:1). The age at onset ranged from 1 month (initial manifestation: cardiac rhabdomyoma) to 13 years (initial manifestation: epilepsy). At first presentation, 35 patients were aged <1 year, 26 were 1–3 years, and 12 were >3 years. Epilepsy was the most common presentation (62/73, 85%), followed by hypopigmented macules (44/73, 60%), cortical tubers (34/73, 47%), intellectual disability (24/73, 33%), subependymal nodules (22/73, 30%), cardiac rhabdomyomas (11/73, 15%), café-au-lait spots (9/73, 12%), facial angiofibromas (4/73, 5%), shagreen patches (4/73, 5%), retinal pigment loss (2/73, 3%), retinal hamartomas (1/73, 1%), frontal fibromas (1/73, 1%), and multiple renal cysts (1/73, 1%) (Table 2). Consistent with the 11 major and 6 minor diagnostic criteria for TSC, hypopigmented macules were the most prevalent cutaneous feature, while other skin lesions were less frequent; seven children had two types of skin lesions. Only one patient presented with the classic Vogt triad (epilepsy, intellectual disability, and facial angiofibromas). Although epilepsy is not a formal diagnostic criterion for TSC, it was the most common clinical manifestation in this cohort (85%), a prevalence consistent with prior reports (1). The presence of intracranial nodules (cortical tubers or subependymal nodules) was most frequently associated with epileptic seizures.

**Table 2:** Clinical features in different groups of mutation types.

Clinical feature	Total (*n* = 73)	TSC1 (*n* = 19)	TSC2 (*n* = 49)	NMI (*n* = 5)	TSC1 vs. TSC2	NMI vs. Mut.
	No.	No.	No.	No.	*P* value	*P* value
Male	46 (63%)	14 (74%)	28 (57%)	4 (80%)	/	/
Female	27 (37%)	5 (26%)	21 (43%)	1 (20%)	/	/
Skin						
Hypomelanotic macules	44 (60%)	12 (63%)	31 (63%)	1 (20%)	ns	0.015
Café-au-lait macules	9 (12%)	1 (5%)	7 (14%)	1 (20%)	ns	ns
Facial angiofibromas	4 (5%)	1 (5%)	3 (6%)	0 (0%)	ns	ns
Shagreen patch	4 (5%)	1 (5%)	3 (6%)	0 (0%)	ns	ns
Fibrous forehead plaque	1 (1%)	1 (5%)	0 (0%)	0 (0%)	ns	ns
CNS						
Epileptic Seizure	62 (85%)	18 (95%)	41 (84%)	3 (60%)	ns	ns
Cortical tubers	34 (47%)	12 (63%)	20 (41%)	2 (40%)	ns	ns
Subependymal nodules	22 (30%)	6 (32%)	14 (29%)	2 (40%)	ns	ns
Intellectual disability	24 (33%)	6 (32%)	15 (31%)	3 (60%)	0.048	ns
Cardiovascular						
Cardiac rhabdomyoma	11 (15%)	2 (11%)	9 (18%)	0 (0%)	ns	ns
Kidney						
Multiple renal cysts	1 (1%)	0 (0%)	1 (2%)	0 (0%)	ns	ns
Ophthalmic						
Retinal hypopigmented patches	2 (3%)	0 (0%)	2 (4%)	0 (0%)	ns	ns
Retinal hamartoma	1(1%)	0(0%)	1(2%)	0(0%)	ns	ns

“Mut.” refers to the mutation-positive group (TSC1 or TSC2).

Only statistically significant *P* values are shown; ns indicates *P* ≥ 0.05.

CNS, central nervous system; NMI, no mutation identified; ns, no statistically significant difference (*P* ≥ 0.05); TSC, tuberous sclerosis complex.

In this cohort of 73 pediatric patients with TSC, the most common initial presenting feature was epilepsy (62/73, 85%). Other presenting signs included hypopigmented macules (7/73, 10%), cardiac rhabdomyomas (2/73, 3%), café-au-lait spots (2/73, 3%), intellectual disability (2/73, 3%), retinal hamartomas (1/73, 1%), limb hypertrophy (1/73, 1%), and headaches (1/73, 1%). Among the 62 patients with epilepsy, seizure types were categorized as follows: epileptic spasms (*n* = 25), generalized tonic-clonic seizure (*n* = 13), focal impaired consciousness seizure (*n* = 10), focal preserved consciousness seizure (*n* = 6), focal to bilateral tonic-clonic seizure (*n* = 5), tonic seizures (*n* = 1), and absence seizures (*n* = 1) (14).

### 3.3 Genotype-phenotype correlation

Genotype-phenotype correlation analysis was performed in 73 patients with TSC. Based on the identified mutations, patients were categorized into three groups: TSC1 (*n* = 19), TSC2 (*n* = 49), and no mutation identified (NMI, *n* = 5). Seizures were observed in all groups (TSC1: 95%; TSC2: 84%; NMI: 60%), with no statistically significant differences among them. However, intellectual disability was significantly more prevalent in the NMI group (60%) than in the mutation-positive groups (TSC1: 32%; TSC2: 31%; *p* = 0.048 for TSC1 vs. TSC2). Conversely, hypopigmented macules were significantly less frequent in the NMI group (20%) than in patients with identified TSC1 or TSC2 mutations (63%; *p* = 0.015). No significant intergroup differences were observed in other cutaneous, neurological, cardiovascular, renal, or ocular manifestations.

### 3.4 Treatment and prognosis

All 62 pediatric TSC patients with seizures received antiepileptic therapy and were followed for treatment regimens and efficacy. The treatments included: monotherapy (18 patients), dual-drug therapy (21 patients), triple-drug therapy (11 patients), sirolimus (with antiepileptic drugs; 9 patients), ACTH pulse therapy (3 patients), the ketogenic diet (2 patients), and surgery combined with antiepileptic drugs (1 patient). Among conventional antiepileptic drugs (AEDs), vigabatrin was the most frequently used (22/62,35%), followed by valproate (20/62,32%), topiramate (17/62,27%), and oxcarbazepine (11/62,18%). Follow-up of the nine patients receiving sirolimus showed that all experienced a reduction in seizure frequency or duration, with no adverse reactions observed and generally high parental satisfaction. In contrast, the two patients on the ketogenic diet showed no significant improvement in seizure control. One patient with epilepsy and intracranial calcifications underwent right anterior temporal lobectomy plus right frontal lesion resection. Postoperatively, the patient was maintained on oxcarbazepine and has remained seizure-free for over two years, with improved follow-up EEG findings.

## 4 Discussion

TSC is a rare autosomal dominant neurocutaneous syndrome. The disease has an incidence rate of approximately 1 in 6,000–1 in 10,000. The current understanding of its pathogenesis involves mutations in the *TSC1/2* genes, which activate the mTOR signaling pathway. This leads to the formation of hamartomas in multiple organs throughout the body, most commonly in the skin, brain, kidneys, lungs, and heart. As normal cellular tissue is replaced by various types of cellular structures, this results in functional abnormalities in the corresponding organs (15).

TSC occurs in both sporadic and familial forms, with approximately two-thirds of cases historically reported as sporadic and one-third as familial. The disorder demonstrates no significant gender or racial predilection (16, 17). This cohort offers a distinct contribution to the existing literature. While several cohorts of patients with TSC have been previously reported in China, most have encompassed a broad age range or concentrated on specific clinical features. In contrast, our study presents a comprehensive, single-center analysis of a relatively large pediatric cohort, all of whom underwent standardized clinical evaluation and whole-exome sequencing. This study involved 73 pediatric patients of Han Chinese ethnicity, comprising 46 males and 27 females (male-to-female ratio 1.70:1). Within this cohort, 30 patients (41%) had a family history of TSC in a first-degree relative. The age at clinical onset ranged from 1 month to 13 years. A plurality of patients (35/73, 48%) presented before one year of age. Advances in prenatal and neonatal screening in China have facilitated the detection of initial manifestations, such as cardiac or retinal lesions, in some patients during the perinatal period. The clinical manifestations of TSC are diverse and follow an age-dependent pattern. Cardiac rhabdomyomas, for example, typically appear prenatally (detectable after 22 weeks of gestation) and often regress spontaneously within the first year of life. Renal angiomyolipomas usually emerge around age 1, while periungual fibromas and pulmonary lymphangioleiomyomatosis are more common in adolescence and adulthood, respectively (18, 19). In this cohort, 11 patients under 1 year of age had cardiac rhabdomyomas. Follow-up of these patients revealed complete resolution of the tumors in all 10 who were over 2 years old at last assessment, consistent with the natural history. Notably, two of these 10 patients had a concomitant atrial septal defect (ASD). One of them underwent successful ASD repair at our institution for symptomatic relief, with resolution of dizziness postoperatively. A causal link between TSC and ASD has not been established.

Epilepsy is the most common clinical manifestation of pediatric TSC, with approximately 90% of affected children experiencing seizures within the first three years of life. Seizure types vary widely, most frequently presenting as infantile spasms (20–23). The incidence of epilepsy in this cohort was 85%, with seizures being the initial presenting complaint in the vast majority, consistent with previous reports. Among the 62 pediatric TSC patients with epilepsy, 25 (40%) presented with convulsive seizures. All patients underwent antiepileptic therapy: 18 received monotherapy and 41 received polytherapy (≥2 AEDs). The most frequently used antiseizure medications were vigabatrin, valproate, topiramate, and oxcarbazepine. Nine patients received adjunctive rapamycin/sirolimus; follow-up showed improved seizure frequency or duration with good tolerability. In contrast, the two patients attempting ketogenic diet showed no significant benefit. One patient with intracranial calcifications remained seizure-free for over 2 years following right anterior temporal and frontal lesion resection while maintained on oxcarbazepine, with corresponding EEG improvement. These findings suggest that mTOR inhibitors or surgery may benefit some pediatric patients refractory to conventional drug therapy, highlighting the value of individualized management.

Multiple intracranial nodules or calcifications are a hallmark central nervous system manifestation in children with TSC and a recognized cause of epilepsy (24). Studies indicate that approximately 75% of epileptic seizures in TSC are associated with these intracranial lesions, which may remain asymptomatic initially. Consequently, many children first present with epilepsy, and the nodules are discovered on neuroimaging. Pathologically, these lesions are classified as cortical/subcortical tubers and subependymal nodules (25). In our cohort of 73 pediatric patients, intracranial nodules were the most common neurological manifestation, identified in 49 cases, and indeed represented the highest prevalence among all TSC-related pathologies. Among these 49 patients, the lesions included cortical nodules (*n* = 34) and subependymal nodules (*n* = 22). Epilepsy co-occurred with intracranial nodules in 49 patients, accounting for 79% of all epilepsy cases in the study, which aligns with prior reports. In one illustrative case, surgical resection of an intracranial lesion led to significant seizure improvement, providing direct evidence of the strong association between the two conditions.

Vogt's triad (epilepsy, facial angiofibromas, and intellectual disability) is a classic TSC phenotype, historically reported in 30%–40% of pediatric patients (26). In the present study, only one child fulfilled all three criteria, a prevalence markedly lower than literature reports. Several factors may account for this difference. Notably, facial angiofibromas were observed in only 4 of our 73 patients, against a reported background frequency of 83%–90% (27, 28). The low frequency of this key component largely explains the rarity of the complete triad, despite epilepsy and intellectual disability being common. Furthermore, facial angiofibromas exhibit age-related onset, usually appearing in early childhood, and some participants were still infants. The mutation spectrum in our cohort also showed limited correlation with this cutaneous phenotype. Finally, the modest sample size may contribute to this observed deviation.

Hypopigmented macules are a classic cutaneous manifestation of TSC. Historically, these lesions were often mistaken for benign “birthmarks,” leading to medical consultation only after the onset of more overt symptoms such as seizures or facial angiofibromas. Among the 73 children, 7 were initially diagnosed based on hypopigmented macules, all before 1 year of age. Overall, 44 patients exhibited this phenotype, a prevalence significantly higher than that of other cutaneous features (e.g., café-au-lait spots or facial angiofibromas) and even exceeding some traditional neurological manifestations such as intellectual disability. This highlights the critical importance of a meticulous physical examination. In particular, TSC should be considered in the differential diagnosis for infants under 1 year of age who present with hypopigmented macules.

While renal angiomyolipoma (AML) incidence in TSC increases with age (≈65% in childhood, up to 80% in adults) (29), none of the 73 pediatric patients in our study presented with AML at diagnosis or on follow-up. This may be attributed to the low prevalence of AML in young children (<12 years) and its typical emergence in adolescence. Additionally, TSC-associated AML exhibits a marked female predominance (male-to-female ratio approximately 1:4) (30), which contrasts with the male-skewed sex distribution in our cohort.

The *TSC1* gene was first localized to 9q34 in 1987 (31), followed by the identification of *TSC2* on 16p13.3 a decade later. Collectively, more than a thousand mutations have been identified in these genes. Among confirmed cases, roughly 70% harbor *TSC2* mutations and 20% harbor *TSC1* mutations. In a minority of patients, no mutation is detected by conventional methods, possibly due to mosaicism, variants in non-exonic regions, or other genetic causes (32–34). Somatic mosaicism results in the restriction of mutations to specific tissues or cell subsets. Routine genetic testing using peripheral blood leukocytes often fails to detect such localized variants, thereby leading to false-negative results. Furthermore, routine coding-region sequencing and hotspot screening fail to detect pathogenic variants in deep intronic regions, promoters, untranslated regions and other regulatory elements, reducing diagnostic yield. In addition, cryptic genetic mechanisms including copy number variations, structural rearrangements, epigenetic modifications and mitochondrial mutations may also cause negative genetic results. However, we acknowledge that in our five patients with NMI, we were unable to perform tissue-based testing (e.g., from skin lesions or cortical tubers) to investigate the possibility of low-level mosaicism. This was due to the retrospective nature of our study and the ethical constraints associated with obtaining such samples from pediatric patients. Therefore, although mosaicism is a plausible explanation for some of these negative results, it could not be definitively confirmed. The *TSC1* gene comprises 23 exons, with hotspots in exons 15, 18, and 21, predominantly harboring nonsense or frameshift mutations. In our cohort, we identified 19 *TSC1* mutations [11 *de novo*, 8 inherited (4 paternal, 4 maternal)], consisting of 9 nonsense, 5 frameshift, 3 missense, and 2 splice-site variants. The *TSC2* gene has 42 exons, with mutational clusters in exons 33, 37, and 40. The spectrum is dominated by missense and nonsense mutations, along with large deletions. Among the 52 *TSC2* mutations we identified, the distribution was as follows: 22 missense, 13 frameshift, 8 splice-site, 6 nonsense, 1 synonymous, and 2 exon deletions. These findings are largely consistent with established mutation patterns. Intellectual disability differed significantly between the *TSC1* and *TSC2* mutation groups (*p* = 0.048). Notably, hypopigmented macules were significantly more frequent in patients with identified mutations (combined *TSC1/TSC2* incidence: 63%) compared to the NMI group (20%) (*p* = 0.015). In contrast, the incidence of hypopigmented macules did not differ between the TSC1 and TSC2 subgroups themselves (63% in each). All other examined clinical features—including additional cutaneous, neurological (non-epileptic), cardiovascular, renal, and ocular findings—showed no statistically significant differences among the TSC1, TSC2, and NMI groups.

It is important to acknowledge that this study employed a retrospective cross-sectional design, and the clinical features reported are based on data obtained at initial diagnosis and routine outpatient follow-up visits. Given the age-dependent nature of TSC manifestations, the absence of standardized longitudinal follow-up may limit the capture of phenotypic evolution over time. Future prospective longitudinal studies are warranted to systematically delineate the natural history and age-related progression of TSC in children.

In summary, this study confirms the broad genotypic and phenotypic heterogeneity of TSC in a pediatric cohort. *TSC2* mutations were more prevalent than *TSC1* mutations and were associated with a greater number of pathogenic variants and more severe clinical manifestations. Although the type and location of mutations appeared to correlate with epilepsy risk and disease severity, the limited sample size precluded definitive genotype-phenotype conclusions. These findings underscore the clinical utility of early molecular genetic testing in suspected TSC cases, facilitating accurate diagnosis, genetic counseling, and informed prenatal planning for affected families. Given the rarity of TSC, larger multicenter collaborations are warranted to validate these observations and further delineate genotype-phenotype relationships. Future studies should also consider integrating tissue-based genetic testing, when clinically and ethically feasible, to elucidate the role of somatic mosaicism—particularly in patients who remain mutation-negative on standard blood-based assays.

## Data Availability

The original contributions presented in the study are included in the article/Supplementary Material, further inquiries can be directed to the corresponding authors.
